# Editorial: Formation and remodeling of immunological niches in tumors: organ-specific mechanisms and inflammatory parallels, volume II

**DOI:** 10.3389/fonc.2026.1923611

**Published:** 2026-08-05

**Authors:** Zilu Chen, Xiaolei Hao, Liang Li, Qingyu Luo, Weiling Li, Ying Luo, Xiaosheng Tan

**Affiliations:** 1 Harvard Medical School, Boston, MA, United States; 2 Department of Immunology, St Jude Children’s Research Hospital, Memphis, TN, United States; 3 Guangdong Provincial People’s Hospital (Guangdong Academy of Medical Sciences), Southern Medical University, Guangzhou, Guangdong, China; 4 AureLume Biosciences Co., Ltd., Hangzhou, Zhejiang, China; 5 Department of Biotechnology, College of Basic Medical Sciences, Dalian Medical University, Dalian, Liaoning, China; 6 Department of Immunology, University of Texas (UT) Southwestern Medical Center, Dallas, TX, United States; 7 Child Health Institute of New Jersey, Rutgers Robert Wood Johnson Medical School, New Brunswick, NJ, United States

**Keywords:** immune remodeling, immunological niches, spatial organisation, tumor immunity, tumor microenvironment

## Introduction

As Shakespeare wrote in Macbeth, “Fair is foul, and foul is fair,” reminding us that the same phenomenon may have markedly different consequences depending on its context. A similar principle is evident in tumor immunity, where comparable immune compositions may give rise to markedly different clinical outcomes depending on the organ and disease setting (1). Increasing evidence suggests that tumor immunity is not defined by immune cell abundance alone, but by the context-specific formation and remodeling of immunological niches, which are spatially and functionally organized microenvironments that shape immune behavior (2, 3). Notably, comparable principles of niche organization and remodeling have been observed in chronic inflammatory and degenerative diseases, pointing to a shared biological logic that transcends disease boundaries (4, 5). Together, these insights suggest that a niche-centric perspective may provide a more comprehensive framework for understanding immune heterogeneity across diseases.

Against this conceptual background, and building on the success of the first volume, the Research Topic Formation and Remodeling of Immunological Niches in Tumors: Organ-Specific Mechanisms and Inflammatory Parallels, Volume II was launched to highlight recent developments in this field. The Research Topic brings together 21 contributions, predominantly original research articles complemented by reviews and an opinion article, providing new insights into the formation, maintenance, and remodeling of immunological niches in solid tumors, with particular emphasis on organ-specific immune architectures and regulatory mechanisms. Collectively, these studies also demonstrate how mechanistic insights from chronic inflammation, neuroimmune interactions, and other tissue-specific inflammatory settings can deepen our understanding of tumor immunity and inspire future therapeutic strategies.

Together, these studies reveal that immunological niches should be understood not as static microenvironments, but as dynamic and context-dependent ecosystems whose formation, remodeling, and functional significance are better understood through integrated spatial, molecular, and cross-disease perspectives. The following sections discuss these contributions from three complementary perspectives.

## Architecting immunological niches in space and context

Recent advances in tumor immunology have increasingly highlighted that immune function is governed not only by cellular composition but also by spatial organization and tissue context (6, 7). Studies within this Research Topic collectively demonstrate that immunological niches arise from coordinated regulatory circuits that couple metabolic reprogramming with immune signaling. Multi-omics analyses in inflammatory settings, such as ulcerative colitis, reveal metabolic–immune regulators linking mitochondrial function to immune activation and tissue integrity (8). Similarly, emerging regulatory paradigms, including liquid–liquid phase separation, further illustrate how molecular organization contributes to tumor heterogeneity, spatial architecture, and immune microenvironment remodeling.

Importantly, these niches are inherently spatially organized. Single-cell transcriptomic profiling of lung adenocarcinoma lymph node metastases identified distinct malignant epithelial subpopulations with unique copy number variation patterns and transcriptional programs. Likewise, single-cell multi-omics analysis of high-risk B-cell acute lymphoblastic leukemia (B-ALL) identified therapy-resistant HSC/MPP and Pro-B cell subpopulations enriched for distinct transcriptional signatures and signaling pathways. In glioblastoma, spatially restricted mesenchymal-like programs driven by immune communication define functionally specialized microenvironments associated with poor prognosis. In Lewis lung carcinoma, tissue-resident immune cells such as alveolar macrophages exhibit compartmentalized localization and function, maintaining distinct immune states between tumor and extra-tumoral regions. Consistent with these findings, pan-cancer analyses of regulators such as TEAD4 further demonstrate that immune cell infiltration, stromal composition, and checkpoint activity are tightly linked to spatially organized transcriptional programs within the tumor microenvironment.

Taken together, these studies suggest that the formation of immunological niches emerges from coordinated spatial organization and molecular regulation, in which tissue architecture and regulatory networks jointly shape immune function.

## Dynamic remodeling and functional plasticity of immune niches

While niche formation establishes the structural foundation of tumor immunity, immunological niches are highly dynamic and undergo continuous remodeling during disease progression and therapeutic intervention (8). Multiple studies demonstrate that tumor-associated genes and signaling pathways modulate immune infiltration, checkpoint expression, and stromal interactions, thereby reshaping the immune landscape in a context-dependent manner. For example, R3HDM4 was identified as a regulator of kidney renal clear cell carcinoma progression and immune modulation, with potential links to the IGSF8 immune checkpoint. MFSD12 was shown to promote hepatocellular carcinoma proliferation, metastasis, and invasion, while exhibiting potential associations with the HAVCR2/LGALS9 immune checkpoint axis. CHMP4A was implicated in hepatocellular carcinoma progression and immune modulation, with a potential connection to TIM3 checkpoint signaling. Furthermore, integrative multi-omics analyses identified BAG5 as a regulator of non-small cell lung cancer progression, providing mechanistic insights into the molecular pathways underlying tumor progression. Likewise, immunometabolic pathways such as ferroptosis emerge as critical regulators of niche remodeling, as illustrated by studies demonstrating that BUB1-mediated STAT3/GPX4 signaling and ferroptosis-driven immune subtypes shape tumor progression and therapeutic response.

At the cellular level, immune states such as T cell exhaustion exemplify the plasticity of niche-dependent regulation. Exhausted T cells exhibit dynamic transitions, epigenetic reprogramming, and partial functional reversibility, all of which are shaped by metabolic stress and microenvironmental constraints. In parallel, analyses of ascitic immune environments in ovarian cancer further highlight how dynamic immune cell states, particularly T cell phenotypes, contribute to prognosis and chemotherapy resistance.

Furthermore, niche remodeling is closely linked to clinical outcomes. Integrative models combining immune, clinical, and inflammatory parameters demonstrate that dynamic immune–inflammatory states significantly influence survival and treatment response in cancers such as small cell lung cancer.

Rather than static entities, immunological niches emerge as adaptive ecosystems whose continuous remodeling integrates tumor-intrinsic programs with microenvironmental cues to shape disease trajectories and therapeutic responses.

## Immunological niches across diseases and therapeutic landscapes

Beyond tumor biology, an emerging theme across this Research Topic is the convergence of immunological niche principles across diverse disease contexts. Notably, several contributions highlight how inflammatory signaling pathways act as central drivers of niche formation and remodeling. For example, neutrophil extracellular traps (NETs) shape thrombo-inflammatory niches in pancreatic ductal adenocarcinoma, linking inflammation, coagulation, and immune regulation. Similarly, activation of the mtDNA-cGAS-STING pathway in autoimmune disease models illustrates how innate immune sensing mechanisms orchestrate inflammatory microenvironments and immune activation. At the same time, pyroptosis-related regulators such as GSDME demonstrate context-dependent roles in shaping inflammatory responses, immune cell infiltration, and therapeutic sensitivity across cancers.

This convergence suggests a shared biological logic underlying niche dynamics across disease contexts. Aging-associated immune remodeling further supports this concept, as tissue-resident T cells acquire distinct transcriptional and functional profiles that contribute to tissue homeostasis and disease susceptibility in brain-associated tissues. In parallel, systemic immune–inflammatory indices and circulating biomarkers provide additional evidence that immune niche states extend beyond local tumor sites and reflect organism-wide immune regulation in lung cancer.

From a translational perspective, these insights support a shift from tumor-centric to niche-centric therapeutic strategies. Approaches such as oncolytic virotherapy demonstrate the potential to actively reprogram immunological niches, transforming immunosuppressive environments into immunostimulatory states and enhancing anti-tumor immunity.

This conceptual convergence positions immunological niche remodeling as a unifying therapeutic entry point, where targeting niche formation, maintenance, and plasticity may enable more effective interventions across both cancer and chronic inflammatory diseases.

Collectively, the studies presented in this Research Topic converge on a central insight: tumor immunity cannot be fully understood through immune cell composition alone, but must be interpreted within the context of spatially organized and dynamically evolving immunological niches. These niches, shaped by tissue-specific architecture and continuously remodeled through metabolic, molecular, and cellular interactions, define the functional states of immune responses across disease contexts. Importantly, the parallels observed between cancer and chronic inflammatory diseases suggest that immunological niche remodeling represents a conserved biological principle rather than a tumor-specific phenomenon. Moving forward, integrating spatial, temporal, and cross-disease perspectives will be essential to decode immune regulation at the systems level. These findings support a niche-centric perspective that may not only reshape our understanding of immune regulation and disease heterogeneity, but also encourage a shift in therapeutic strategies from targeting individual components toward reprogramming the immune ecosystem as an integrated system.
